# Multidimensional Sexual Perfectionism and Female Sexual Function: A Longitudinal Investigation

**DOI:** 10.1007/s10508-016-0721-7

**Published:** 2016-03-28

**Authors:** Joachim Stoeber, Laura N. Harvey

**Affiliations:** School of Psychology, University of Kent, Canterbury, Kent CT2 7NP UK

**Keywords:** Perfectionism, Sexuality, Sexual self-concept, Female sexual function, Longitudinal analyses

## Abstract

Research on multidimensional sexual perfectionism differentiates four forms: self-oriented, partner-oriented, partner-prescribed, and socially prescribed. Self-oriented sexual perfectionism reflects perfectionistic standards people apply to themselves as sexual partners; partner-oriented sexual perfectionism reflects perfectionistic standards people apply to their sexual partner; partner-prescribed sexual perfectionism reflects people’s beliefs that their sexual partner imposes perfectionistic standards on them; and socially prescribed sexual perfectionism reflects people’s beliefs that society imposes such standards on them. Previous studies found partner-prescribed and socially prescribed sexual perfectionism to be maladaptive forms of sexual perfectionism associated with a negative sexual self-concept and problematic sexual behaviors, but only examined cross-sectional relationships. The present article presents the first longitudinal study examining whether multidimensional sexual perfectionism predicts changes in sexual self-concept and sexual function over time. A total of 366 women aged 17–69 years completed measures of multidimensional sexual perfectionism, sexual esteem, sexual anxiety, sexual problem self-blame, and sexual function (cross-sectional data). Three to six months later, 164 of the women completed the same measures again (longitudinal data). Across analyses, partner-prescribed sexual perfectionism emerged as the most maladaptive form of sexual perfectionism. In the cross-sectional data, partner-prescribed sexual perfectionism showed positive relationships with sexual anxiety, sexual problem self-blame, and intercourse pain, and negative relationships with sexual esteem, desire, arousal, lubrication, and orgasmic function. In the longitudinal data, partner-prescribed sexual perfectionism predicted increases in sexual anxiety and decreases in sexual esteem, arousal, and lubrication over time. The findings suggest that partner-prescribed sexual perfectionism contributes to women’s negative sexual self-concept and female sexual dysfunction.

## Introduction

Perfectionism is characterized by striving for flawlessness and setting exceedingly high standards for performance accompanied by tendencies for overly critical self-evaluations and concerns about negative evaluations by others (Flett & Hewitt, 2002; Frost, Marten, Lahart, & Rosenblate, 1990). Perfectionism is a common personality characteristic that may affect all domains of life (Stoeber & Stoeber, 2009) including people’s sex life (Habke, Hewitt, & Flett, 1999; Snell & Rigdon, 2001; Stoeber, Harvey, Almeida, & Lyons, 2013). The longitudinal consequences of how perfectionism affects people’s sex life, however, are yet unexplored. The aim of the present research was to present a first exploration of these consequences.

Early theory and research on sexual perfectionism—that is, perfectionism focused on sexuality—followed a unidimensional conception of perfectionism (Eidelson & Epstein, 1982; Quadland, 1980). In the 1990s, however, researchers recognized that perfectionism comes in different forms and is therefore best conceptualized as a multidimensional characteristic (Frost et al., 1990; Hewitt & Flett, 1991; see also Enns & Cox, 2002). This is important as the various dimensions of perfectionism have shown different, sometimes opposite relationships with indicators of psychological well-being and psychological maladjustment (Frost, Heimberg, Holt, Mattia, & Neubauer, 1993; see Stoeber & Otto, 2006, for a review). The same applies to sexual perfectionism regarding indicators of sexual well-being and sexual maladjustment (Snell, 2001; Snell & Rigdon, 2001; Stoeber et al., 2013). Consequently, sexual perfectionism is also best conceptualized as a multidimensional characteristic.

### Multidimensional Sexual Perfectionism

According to Hewitt and Flett’s (1991) influential model of multidimensional perfectionism, perfectionism has personal and interpersonal aspects. Consequently, three basic forms of general perfectionism need to be differentiated: self-oriented, other-oriented, and socially prescribed. Self-oriented perfectionism reflects beliefs that striving for perfection and being perfect are important. Self-oriented perfectionists expect themselves to be perfect. Other-oriented perfectionism reflects beliefs that it is important for others to strive for perfection and be perfect. Other-oriented perfectionists expect others to be perfect. In contrast, socially prescribed perfectionism reflects beliefs that striving for perfection and being perfect are important to others. Socially prescribed perfectionists believe that others expect them to be perfect.

In numerous studies across different research groups, the three forms of perfectionism have shown different patterns of relationships with personality characteristics, psychological processes, and key indicators of psychological adjustment and maladjustment (see, e.g., Hewitt & Flett, 2004). Overall, the results suggest that only socially prescribed perfectionism is a purely maladaptive form of perfectionism that is consistently associated with negative characteristics, dysfunctional processes, and indicators of psychological maladjustment. In comparison, self-oriented and other-oriented perfectionism are mixed adaptive–maladaptive forms of perfectionism that are often associated with negative characteristics, dysfunctional processes, and indicators of psychological maladjustment but may also show positive relationships with positive characteristics, functional processes, and indicators of psychological adjustment (for reviews, see Enns & Cox, 2002; Hewitt & Flett, 2004; Stoeber, 2014a, 2014b; Stoeber et al., 2013).

Based on Hewitt and Flett’s (1991) multidimensional model of perfectionism, Snell (1997) developed a multidimensional model of *sexual* perfectionism differentiating four forms: self-oriented, partner-oriented, partner-prescribed, and socially prescribed. Self-oriented sexual perfectionism reflects perfectionistic standards and expectations that people apply to themselves as sexual partners (e.g., “I have very high perfectionistic goals for myself as a sexual partner”). Partner-oriented sexual perfectionism is other-oriented perfectionism applied to one’s sexual partner and reflects perfectionistic standards and expectations that people apply to their partner (e.g., “I expect nothing less than perfection from my sexual partner”). Partner-prescribed sexual perfectionism is socially prescribed perfectionism applied to one’s sexual partner and reflects people’s beliefs that their partner imposes perfectionistic standards and expectations on them (e.g., “My partner demands nothing less than perfection of me as a sexual partner”). In comparison, socially prescribed sexual perfectionism reflects people’s beliefs that society and people in general impose perfectionistic sexual standards and expectations on them (“Most people in society expect me to always be a perfect sexual partner”).^1^ Note that the latter two forms reflect subjective beliefs, not veridical perceptions of others’ actual expectations. People high in partner-prescribed and socially prescribed sexual perfectionism *believe* that others (i.e., their partner, society) expect them to be perfect sexual partners.

To date, there have been three published studies investigating multidimensional sexual perfectionism following Snell’s (1997) model: Snell and Rigdon (2001), Snell (2001), and Stoeber et al. (2013). The first study (Snell & Rigdon, 2001) examined male and female university students. All four forms of sexual perfectionism showed positive correlations with sexual monitoring (concern with others’ impressions of one’s sexuality) in female students. Self-oriented and partner-oriented sexual perfectionism, however, also showed positive correlations with sexual assertiveness (acting in an independent, self-reliant fashion concerning one’s sexuality). Furthermore, partner-oriented sexual perfectionism showed positive correlations with sex-appeal consciousness (alertness to others’ perception that one is “sexy”) in male and female students. The second study (Snell, 2001) examined female university students and found self-oriented sexual perfectionism was positively correlated with feeling comfortable and satisfied with one-night stands. In contrast, partner-prescribed and socially prescribed sexual perfectionism showed positive correlations with feeling guilty after sex. The same held for partner-oriented sexual perfectionism. Moreover, all four forms of sexual perfectionism were associated with problematic attachment styles as indicated by positive correlations with fearful and dismissing attachment and negative correlations with secure attachment.

The third study (Stoeber et al., 2013) also examined male and female university students, but did not analyze the data separately for male and female students. However, the study went beyond examining bivariate correlations and conducted multiple regression analyses controlling for the overlap between the four forms of perfectionism (which have shown large-sized positive intercorrelations) to examine their unique relationships. Results showed that the four forms of sexual perfectionism displayed different patterns of unique relationships. Self-oriented sexual perfectionism showed positive relationships with sexual esteem, sexual self-efficacy, and sex life satisfaction and a negative relationship with sexuality-related depression. However, it also showed a positive relationship with concern over mistakes during sex. Partner-oriented sexual perfectionism showed positive relationships with sexual esteem, sexual self-efficacy, and sexual optimism, a negative relationships with sexual anxiety, and—differently from self-oriented sexual perfectionism—a negative relationship with concern over mistakes during sex. In contrast, partner-oriented and socially prescribed sexual perfectionism showed relationships indicative of sexual maladjustment. Partner-prescribed sexual perfectionism showed a positive relationship with sexual problem self-blame; and socially prescribed sexual perfectionism showed positive relationships with sexual anxiety, sexuality-related depression, and concern over mistakes during sex and negative relationships with sexual esteem and sexual optimism. Taken together, the findings of the three studies suggest that self-oriented and other-oriented sexual perfectionism are mixed adaptive–maladaptive forms of sexual perfectionism showing positive and negative relationships with indicators of a negative sexual self-concept and problematic sexual behaviors. In contrast, partner-prescribed and socially prescribed sexual perfectionism are purely maladaptive forms of sexual perfectionism showing only positive relationships with these indicators.

### The Present Study

As the first systematic investigation examining the unique relationships of the four forms of sexual perfectionism with a range of positive and negative indicators of sexual self-concept, Stoeber et al.’s (2013) study represents an important step forward in our understanding of multidimensional sexual perfectionism. Nevertheless, like the two previous studies (Snell, 2001; Snell & Rigdon, 2001), the study had a number of limitations. First, the study only included university students. Consequently, the findings may not be representative for adults who are older or non-student populations. Second, the study was cross-sectional. Hence, the study was unable to examine whether sexual perfectionism shows any longitudinal relationships with people’s sexual self-concept, for example, predict longitudinal increases or decreases in positive and negative indicators of sexual self-concept. Finally, whereas the study included a measure of sex life satisfaction, it did not examine other aspects of sexual function and dysfunction.

According to the DSM-5 (American Psychiatric Association, 2013), sexual dysfunctions are characterized by significant disturbances in a person’s ability to respond sexually or experience sexual pleasure. Large-scale studies suggest that around 45–50 % of women have experienced problems with sexual function (Mitchell et al., 2013; Shifren, Monz, Russo, Segreti, & Johannes, 2008). At present, however, research on perfectionism and sexual function is limited to two studies examining sexual dysfunction in men. Quadland (1980) investigated the relationship between perfectionism and male sexual dysfunction using a unidimensional measure of sexuality-related perfectionistic thinking. He compared men seeking treatment for erectile dysfunction with a male control group, and found the men seeking treatment to show higher levels of perfectionistic thinking than the control group. DiBartolo and Barlow (1996) examined the relationship of perfectionism and male sexual function in a sample of men diagnosed with erectile dysfunction. They used a multidimensional measure of general perfectionism, but unfortunately examined only overall perfectionism (combining all dimensions to a total perfectionism score). Results showed a positive correlation between overall perfectionism and clinicians’ ratings of the degree to which the men’s erectile difficulties were attributed to psychogenic (rather than organic) factors. No study so far has investigated multidimensional sexual perfectionism and female sexual function. In addition, no study has explored the longitudinal relationships of multidimensional sexual perfectionism.

Against this background, the primary aim of the present study was to provide a first investigation of whether multidimensional sexual perfectionism predicts longitudinal changes in women’s sexual self-concept and female sexual function. To this aim, the study employed a longitudinal correlational design with two measurement points (Taris, 2000) to examine whether multidimensional sexual perfectionism predicts changes in sexual self-concept and sexual function over time. In addition, the study aimed to reinvestigate Stoeber et al.’s (2013) findings regarding the cross-sectional relationships of multidimensional sexual perfectionism with three indicators of sexual self-concept (sexual esteem as a positive indicator, sexual anxiety and sexual problem self-blame as negative indicators) that had shown unique relationships with the different forms of sexual perfectionism. Furthermore, the study sought to examine a sample of female university students and a sample of women recruited over the Internet to provide for an overall older and more representative sample (Gosling, Sandy, John, & Potter, 2010) than the student-only samples of the previous studies on multidimensional sexual perfectionism.

In line with previous findings on multidimensional sexual perfectionism (Snell, 2001; Snell & Rigdon, 2001; Stoeber et al., 2013), we expected the four forms of sexual perfectionism to show different patterns of cross-sectional and longitudinal relationships that could be considered adaptive (positive relationships with variables indicative of a positive sexual self-concept and a higher level of sexual function, negative relationships with variables indicative of a negative sexual self-concept and a lower level of sexual function) or maladaptive (positive relationships with variables indicative of a negative sexual self-concept and a lower level of sexual function, negative relationships with variables indicative of a positive sexual self-concept and a higher level of sexual function). In particular, we expected self-oriented and partner-oriented sexual perfectionism to show mixed adaptive–maladaptive relationships. In contrast, we expected partner-prescribed and socially prescribed sexual perfectionism to show maladaptive relationships only. Apart from these general expectations the study was largely exploratory. Because the research literature on multidimensional sexual perfectionism is still very limited, we did not have specific expectations about what unique relationships the different forms of sexual perfectionism would show with specific aspects of sexual self-concept and sexual function.

## Method

### Participants and Procedure

Two samples of women were recruited to participate in the study: a sample of students from the University of Kent (Sample 1) and a sample from the Internet (Sample 2, consecutively referred to as “Internet users”). Sample 1 was recruited via the School of Psychology’s research participation scheme and through posters distributed around the university. Sample 2 was recruited via the Internet through postings on various research and social networking websites (e.g., Facebook, In-Mind, Online Psychology Research, Psychological Research on the Net, and Twitter). In all recruitments, the study was announced as an online survey investigating whether “personal and interpersonal expectations and beliefs affect one’s sexuality and sexual function.” Furthermore, the study was announced as a two-part study with two measurement points (Time 1 [T1], Time 2 [T2]) requiring participants to provide an email address so they could be contacted for a follow-up survey at T2. All participants completed the survey on the School’s secure Qualtrics^®^ website which required participants to respond to all items on each page before they could move to the next page to avoid missing data. Students who participated received extra course credit or a raffle for one of three £25 (~US $39) vouchers. Internet users received no compensation. The study was approved by the School’s ethic committee and followed the British Psychological Society’s (2009) code of ethics and conduct.

Overall, 366 women responded and completed the T1 survey (December 2013–February 2014): 230 students and 136 Internet users. The mean age of students was 19.7 years (*SD* = 3.4; range: 17–49 years), and the mean age of Internet users 30.0 years (*SD* = 9.2; range = 17–69 years). Of the women, 63.1 % currently had a partner (casual or committed relationship, cohabitation, or married/partnered) and 36.9 % were single. Asked about what sexual orientation described them best, 80.9 % responded “heterosexual,” 12.8 % “bisexual,” 3.3 % “lesbian,” and 3.0 % “questioning.” Using the categories from the university’s equal opportunity monitoring form, participants indicated their ethnicity as White (72.7 %), Black (9.6 %), Asian (5.2 %), mixed race (4.4 %), and other (6.3 %).

After 12 weeks, participants were contacted via email and invited to complete the T2 part of the survey on the School’s Qualtrics^®^ website (May–June 2014). Students who participated received extra course credit or a raffle for one of three £50 (~US $78) vouchers. Internet users received no compensation. Overall, 166 women responded and completed the T2 survey (45 % response rate)—48 students (21 % response rate) and 86 Internet users (63 % response rate)—with T1–T2 intervals ranging from 12.2 to 26.7 weeks (*M* = 17.4, *SD* = 2.0) which corresponds to approximately 3–6 months. Because more women from the Internet sample completed T2 than from the student sample, women completing T2 were significantly older (*M* = 24.2, *SD* = 8.5, range = 17–69 years) than women not completing T2 (*M* = 21.7, *SD* = 6.1, range = 17–54 years), *t*(364) = 3.36, *p* < .001. Consequently, we controlled for sample (student sample vs. Internet sample) and age in all regression analyses (see also “Preliminary Analyses” section).

### Measures

#### Sexual Perfectionism

To measure sexual perfectionism, we used the Multidimensional Sexual Perfectionism Questionnaire (Snell, 1997; see Appendix of Stoeber et al., 2013) capturing self-oriented sexual perfectionism (6 items; e.g., “I have very high perfectionistic goals for myself as a sexual partner”), partner-oriented sexual perfectionism (6 items; “I expect nothing less than perfection from my sexual partner”), partner-prescribed sexual perfectionism (6 items; “My partner demands nothing less than perfection of me as a sexual partner”), and socially prescribed sexual perfectionism (6 items; “Most people in society expect me to always be a perfect sexual partner”). Participants responded to all items on a 5-point scale from 0 (*disagree*) to 4 (*completely agree*), and subscale scores were computed by summing responses across items.

#### Sexual Self-Concept

To measure sexual esteem, we used the sexual esteem subscale from the Sexuality Scale (Snell & Papini, 1989; 10 items; e.g., “I would rate my sexual skill quite highly”); to measure sexual anxiety, we used the sexual anxiety subscale from the Multidimensional Sexual Self-Concept Questionnaire (MSSCQ; Snell, 2011; 4 items; “I feel anxious when I think about the sexual aspects of my life”); and to measure sexual problem self-blame, we used the sexual problem self-blame subscale from the MSSCQ (5 items; “I would be to blame if the sexual aspects of my life were not going very well”). All scales have demonstrated reliability and validity in previous studies (e.g., Snell & Papini, 1989; Stoeber et al., 2013). Participants responded to all items on a 5-point scale from 0 (*not at all characteristic of me*) to 4 (*very characteristic of me*), and scale scores were computed by summing responses across items.

#### Female Sexual Function

To measure female sexual function, we used the Female Sexual Function Index (FSFI; Rosen et al., 2000). The FSFI is the most widely used self-report measure of female sexual function using a four-week timeframe to capture six aspects of sexual function: desire (2 items; e.g., “Over the past 4 weeks, how often did you feel sexual desire or interest? “), arousal (4 items; “Over the past 4 weeks, how often did you feel sexually aroused [“turned on”] during sexual activity or intercourse?”), lubrication (4 items; “Over the past 4 weeks, how often did you become lubricated [“wet”] during sexual activity or intercourse?”), orgasm (3 items; “Over the past 4 weeks, when you had sexual stimulation or intercourse, how often did you reach orgasm?”), satisfaction (3 items; “Over the past 4 weeks, how satisfied have you been with your sexual relationship with your partner?”), and pain (3 items; “Over the past 4 weeks, how often did you experience discomfort or pain during vaginal penetration?”).

The original answer format of the FSFI requires participants to respond to all items on five-point scales with different categories for each item (e.g., from 1 [*almost never or never*] to 5 [*almost always of always*] for the desire items). In addition, all items—except the desire items and two of the satisfaction items—have a response category indicating no sexual activity (0 [*no sexual activity*]) or, in the case of the pain items, no attempted intercourse (0 [*Did not attempt intercourse*]). This format presents two problems (Meyer-Bahlburg & Dolezal, 2007). First, whereas the two desire items do not need a zero category (people can experience desire without sexual activity or intercourse), all three satisfaction items concern *sexual* satisfaction so it is confusing that only one item has a zero category. Following Meyer-Bahlburg and Dolezal, we therefore presented all three satisfaction items with a zero response category (0 [*no sexual activity*]). Second, the original scoring procedure of the FSFI includes zero responses when calculating sum scores for the different subscales. As a consequence, women who had no sexual activity or intercourse over the past four weeks (and therefore score 0 on all items including a zero category) obtain FSFI scores suggesting that they have lower sexual function than women who had sexual activity or intercourse but give their sexual function the lowest rating (i.e., 1 on all items except the pain items). Following Meyer-Bahlburg and Dolezal, we therefore treated all zero responses as missing values and did not compute scores for women who indicated no sexual activity or no attempted intercourse for the respective subscales which was the case for between 14.5 % (orgasm at T1) and 27.0 % (pain at T1) of the sample (see *N*s in Table 1 and table note). Third, in the original FSFI, higher pain scores indicate less pain which may cause problems when interpreting results. Consequently, we reversed the scoring of the pain scale such that higher scores indicated more pain. Otherwise, we followed the original scoring system and computed weighted subscale scores (see appendix of Rosen et al., 2000), that is, subscale scores were computed by summing across items and the resulting scores were then multiplied by 0.6 (desire), 0.3 (arousal, lubrication), or 0.4 (orgasm, satisfaction, pain).

**Table 1:** Descriptive statistics and bivariate correlations

Variable					Correlation			
	*M*	*SD*	*α*	*N*	1	2	3	4
*T1 sexual perfectionism*								
1. Self-oriented	12.43	6.09	.87	366				
2. Partner-oriented	7.36	5.37	.86	366	.53***			
3. Partner-prescribed	7.64	5.30	.85	366	.54***	.67***		
4. Socially prescribed	8.06	5.67	.86	366	.68***	.50***	.62***	
*T2 sexual perfectionism*								
Self-oriented	12.29	5.57	.85	164	.57***	.33***	.36***	.37***
Partner-oriented	7.16	5.06	.87	164	.29***	.65***	.40***	.16*
Partner-prescribed	7.50	5.14	.86	164	.28***	.36***	.63***	.22**
Socially prescribed	8.63	5.50	.87	164	.46***	.22**	.40***	.64***
*T1 dependent variable*								
Sexual self-concept								
Sexual esteem	23.00	8.47	.91	366	.07	.16**	−.06	−.08
Sexual anxiety	4.87	4.69	.94	366	.17**	.10*	.28***	.28***
Sexual problem self-blame	7.59	5.74	.90	366	.33***	.22***	.33***	.36***
Female sexual function								
Desire	3.99	1.20	.90	366	.26***	.12*	.02	.11*
Arousal	4.59	1.07	.88	310	.11	−.11*	−.18**	−.05
Lubrication	5.18	0.92	.79	309	−.01	−.16**	−.22***	−.13*
Orgasm	4.10	1.49	.87	312	−.08	−.12*	−.17**	−.12*
Satisfaction	4.53	1.30	.87	289	−.16**	−.31***	−.30***	−.22***
Pain	2.09	1.06	.88	267	.06	.09	.21***	.15*
*T2 dependent variable*								
Sexual self-concept								
Sexual esteem	22.54	9.08	.94	164	−.03	.15	−.18*	−.12
Sexual anxiety	5.40	5.06	.96	164	.22**	.12	.35***	.32***
Sexual problem self-blame	7.67	5.78	.92	164	.23**	.12	.29***	.26***
Female sexual function								
Desire	3.99	1.16	.89	164	.13	.11	−.05	−.02
Arousal	4.42	1.21	.91	140	.02	−.06	−.28***	−.11
Lubrication	5.10	1.05	.87	140	.05	.04	−.23**	−.05
Orgasm	4.12	1.57	.91	139	−.01	.04	−.15	−.17*
Satisfaction	4.42	1.32	.88	133	−.09	−.22*	−.17*	−.13
Pain	2.19	1.25	.93	121	.09	−.06	.07	.12

T1 = Time 1, T2 = Time 2 (3–6 months later). *α* = Cronbach’s alpha. *N* = number of women. Women reporting no recent sexual activity or intercourse at T1/T2: arousal (*n* = 56/24), lubrication (*n* = 57/24), orgasm (*n* = 54/25), satisfaction (*n* = 77/31), pain (*n* = 99/43). Higher pain scores indicate more pain. (The full correlation matrix is available from the first author.)

* p < .05; ** *p* < .01; *** p < .001

#### Reliability of Measures

We examined the reliability (internal consistency) of all measures by computing Cronbach’s alphas. As Table 1 shows, all the measures showed satisfactory alphas (*α*s ≥ .79).

### Data Analysis

To analyze the data and examine the cross-sectional and longitudinal relationships related to the aims of our study, we employed the following analytic strategy. First, we screened the data for (a) potential differences between participants who completed the T2 survey and those who did not, (b) longitudinal mean changes in the variables, (c) correlations with age, (d) differences between women who had a partner and women who had no partner, and (e) differences between the two samples (see “Preliminary Analyses” section). Next, we analyzed the cross-sectional relationships that multidimensional sexual perfectionism showed at T1, first examining the bivariate correlations of the four forms of sexual perfectionism (see “Cross-Sectional Analyses 1: Correlations” section) and then examining their unique relationships controlling for the overlap between the four forms by means of multiple regression analyses (see “Cross-Sectional Analyses 2: Regressions” section). Finally, we examined whether the four forms of sexual perfectionism at T1 predicted longitudinal changes in sexual self-concept and female sexual function from T1 to T2 by means of multiple regression analyses (see “Longitudinal Analyses: Regressions T1–T2” section). Furthermore, additional analyses that reviewers recommended regarding previous versions of this article were performed (see “Additional Analyses” section). All data analyses were conducted with IBM SPSS^®^ Version 21.

## Results

### Preliminary Analyses

First, we examined whether participants who responded to the invitation and completed the T2 survey (*n* = 166 “responders”) differed from participants who did not respond and complete T2 (*n* = 202 “non-responders”) with respect to the T1 measures. When inspecting the means using *t* tests, only partner-oriented and partner-prescribed sexual perfectionism at T1 showed significant (*p* < .05) differences. Responders reported lower partner-oriented and partner-prescribed sexual perfectionism at T1 than non-responders. Next, we examined if any variables showed mean changes from T1 to T2 using repeated-measures ANOVAs. Only partner-prescribed sexual perfectionism and arousal showed significant changes with participants reporting lower partner-prescribed sexual perfectionism and arousal at T2 compared to T1. Finally, we examined whether age showed significant correlations with any measures. Age showed negative correlations with all four forms of sexual perfectionism at T1 which is in line with findings on age and general perfectionism (Landa & Bybee, 2007; Stoeber & Stoeber, 2009). Moreover, age showed negative correlations with pain at T1 and T2, and a positive correlation with orgasm at T1. Furthermore, using *t* tests, we examined whether there were significant mean differences (a) between the two samples and (b) between women who currently had a partner and women who did not. As regards (a), the Internet sample reported higher sexual esteem at T1 and T2, lower partner-oriented, partner-prescribed, and socially prescribed sexual perfectionism at T1, and lower pain at T1 than the student sample. As regards (b), women who had a partner reported lower sexual perfectionism (all four forms), sexual anxiety, and sexual problem self-blame and higher sexual esteem, desire, arousal, lubrication, orgasm, and satisfaction at T1 and higher self-esteem and satisfaction at T2 than women who had no partner.^2^ Consequently, we controlled for age, sample, and relationship status (i.e., whether women had a partner at T1 or not) in all regression analyses (see Tables 2 and 3).

**Table 2:** T1 multiple regressions: T1 sexual perfectionism predicting T1 dependent variable

	T1 sexual perfectionism				
T1 dependent variable	Self-oriented *β*	Partner-oriented *β*	Partner-prescribed *β*	Socially prescribed *β*	∆*R* ^2^
*Sexual self-concept*					
Sexual esteem	.15*	.38***	−.20**	−.20**	.11***
Sexual anxiety	−.02	−.20**	.25***	.19**	.09***
Sexual problem self-blame	.15*	−.08	.17*	.18*	.14***
*Female sexual function*					
Desire	.31***	.12	−.13	−.03	.09***
Arousal	.32***	−.05	−.22**	−.07	.07***
Lubrication	.18*	−.05	−.18*	−.09	.04**
Orgasm	.03	.03	−.16	−.01	.02
Satisfaction	.05	−.16*	−.15	−.01	.06***
Pain	−.10	−.10	.25**	.11	.04*

*N*s = 267–366 women (cf. Table 1). T1 = Time 1. All multiple regressions controlled for sample and age. *β* = standardized regression coefficient. ∆*R* ^2^ = percentage of variance explained by T1 sexual perfectionism after controlling for sample, age, and relationship status (i.e., whether women had a partner at T1 or not). Higher pain scores indicate more pain

* p < .05; ** *p* < .01; *** p < .001

**Table 3:** T1–T2 multiple regressions: T1 sexual perfectionism predicting T2 dependent variable (DV) controlling for T1 DV

		T1 sexual perfectionism				
T2 dependent variable (DV)	T1 DV *β*	Self-oriented *β*	Partner-oriented *β*	Partner-prescribed *β*	Socially prescribed *β*	∆*R* ^2^
*Sexual self-concept*						
Sexual esteem	.75***	−.09	.11	−.21**	.09	.03*
Sexual anxiety	.65***	.04	−.03	.20**	.00	.04*
Sexual problem self-blame	.59***	.09	−.12	.15	−.09	.02
*Female sexual function*						
Desire	.51***	.10	.05	−.14	−.10	.02
Arousal	.57***	.08	.06	−.22*	−.06	.04
Lubrication	.61***	.04	.13	−.26**	.05	.04
Orgasm	.73***	.11	.06	−.01	−.15	.02
Satisfaction	.36***	.07	−.11	.01	−.12	.02
Pain	.69***	.04	−.03	.03	.03	.00

*N*s = 121–164 women (cf. Table 1). T1 = Time 1, T2 = Time 2 (3–6 months later). All multiple regressions controlled for sample and age. *β* = standardized regression coefficient. ∆*R* ^2^ = percentage of variance explained by T1 sexual perfectionism after controlling for sample, age, and relationship status (i.e., whether women had a partner at T1 or not). Higher pain scores indicate more pain

* *p* < .05; ** p < .01; *** p < .001

### Cross-Sectional Analyses 1: Correlations

Next, we computed bivariate correlations to examine whether the four forms of sexual perfectionism showed different relationships with sexual self-concept and female sexual function at T1 (see Table 1). All forms of sexual perfectionism showed positive correlations with sexual anxiety and sexual problem self-blame, and negative correlations with satisfaction. Else, they showed different correlations. Self-oriented sexual perfectionism showed a positive correlation with desire. Partner-oriented sexual perfectionism showed a positive correlation with sexual esteem and desire, and a negative correlation with arousal, lubrication, and orgasm. Partner-prescribed sexual perfectionism showed a positive correlation with pain, and a negative correlation with arousal, lubrication, and orgasm. Socially prescribed sexual perfectionism showed a positive correlation with desire and pain, and a negative correlation with lubrication and orgasm (but not with arousal).

### Cross-Sectional Analyses 2: T1 Regressions

Because the four forms of sexual perfectionism displayed large-sized intercorrelations (see Table 1), we computed multiple regressions statistically controlling for their overlap to examine the unique relationships that the forms showed with sexual self-concept and female sexual function at T1. For this, we conducted a hierarchical regression analysis on each of the dependent variables at T1 (Cohen, Cohen, West, & Aiken, 2003). The analyses comprised two steps. In Step 1, we entered age, sample, and relationship status as control variables. In Step 2, we simultaneously entered the four forms of sexual perfectionism as predictors. Table 2 shows the results of Step 2 (omitting the effects of the control variables to reduce the table’s complexity).

As expected, self-oriented and partner-oriented sexual perfectionism showed unique relationships that could be considered mixed adaptive–maladaptive. Self-oriented sexual perfectionism showed positive relationships with sexual esteem and three indicators of female sexual function (desire, arousal, lubrication), but also a positive relationship with sexual problem self-blame. Partner-oriented sexual perfectionism showed a positive relationship with sexual esteem and a negative relationship with sexual anxiety, but also a negative relationship with satisfaction.

In contrast, partner-prescribed and socially prescribed sexual perfectionism showed unique relationships that could only be considered maladaptive. Partner-prescribed sexual perfectionism showed a negative relationship with sexual esteem and a positive relationship with sexual anxiety. Furthermore, it showed negative relationships with arousal and lubrication and a positive relationship with pain. Socially prescribed sexual perfectionism showed a negative relationship with sexual esteem and positive relationships with sexual anxiety and sexual problem self-blame, but no significant relationships with any indicators of sexual function.

### Longitudinal Analyses: T1–T2 Regressions

Finally, we examined whether the four forms of sexual perfectionism at T1 predicted longitudinal changes in sexual self-concept and female sexual function from T1 to T2. For this, we conducted hierarchical regression analyses on each of the dependent variables at T2 examining the effects of sexual perfectionism at T1 while including the dependent variable at T1 in the equation (as a so-called “autoregressor”; Taris, 2000) to examine if sexual perfectionism at T1 predicted residual changes in the dependent variables from T1 to T2. As before, we controlled for age, sample, and relationship status. Therefore, the analyses comprised three steps. In Step 1, we entered age, sample, and relationship status as control variables. In Step 2, we entered the dependent variable at T1 as predictor (autoregressor). In Step 3, we simultaneously entered the four forms of sexual perfectionism at T1 as predictors.

Table 3 shows the results of Step 3 (omitting again the effects of the control variables). Partner-prescribed sexual perfectionism was the only form of sexual perfectionism predicting longitudinal changes in sexual self-concept and female sexual function. Regarding sexual self-concept, partner-prescribed sexual perfectionism predicted decreases in sexual self-esteem and increases in sexual anxiety. Regarding female sexual function, partner-prescribed sexual perfectionism predicted decreases in arousal and lubrication.

### Additional Analyses

As additional analyses, we computed moderated regression analyses (Aiken & West, 1991) to examine whether relationship status (i.e., whether women had a partner at T1 or not) moderated the findings shown in Table 2 and 3. Regarding the T1 regression analyses (Table 2), two interactions were significant: self-oriented sexual perfectionism × relationships status on arousal, *t*(298) = −.3.21, *p* < .01; and socially prescribed sexual perfectionism × relationship status on satisfaction, *t*(277) = −2.14, *p* < .05. To further examine these interactions, we conducted simple slopes analyses following the procedures in Frazier, Tix, and Barron (2004). As regards the first interaction, results showed that self-oriented sexual perfectionism had a larger positive regression coefficient on arousal in women who had no partner (*β* = .80, *p* < .001) than in women who had a partner (*β* = .19, *p* < .05). As regards the second interaction, socially prescribed sexual perfectionism had a nonsignificant positive regression coefficient on satisfaction in women who had no partner (*β* = .32, *p* = .066) and a nonsignificant negative coefficient in women who had a partner (*β* = −.10, *p* = .273). Regarding the T1–T2 regression analyses (Table 3), none of the interactions with relationship status was significant.

Finally, we examined whether sexual self-concept and female sexual function at T1 predicted longitudinal changes in sexual perfectionism from T1 to T2 controlling for sexual perfectionism at T2. The only variables with significant regression coefficients were relationship status and sexual problem self-blame at T1 showing positive coefficients on socially prescribed sexual perfectionism at T2 (*β* = .15, *p* < .05, and *β* = .20, *p* < .01, respectively). Women who had a partner and women with high levels of sexual problem self-blame at T1 showed increases in socially prescribed sexual perfectionism from T1 to T2 relative to women who had no partner and women with low levels of sexual problem self-blame.

## Discussion

The primary aim of the present study was to investigate whether multidimensional sexual perfectionism predicts longitudinal changes in women’s sexual self-concept and sexual function. Examining a sample of women aged 17–69 years using a two-wave longitudinal correlational design, we found partner-prescribed sexual perfectionism to predict decreases in sexual self-esteem and increases in sexual anxiety over a period of three to six months. Moreover, partner-prescribed sexual perfectionism predicted reduced sexual function regarding arousal and lubrication. With this, our findings suggest that partner-prescribed sexual perfectionism is a psychological factor that may contribute to problems with female sexual function.

Regarding the stages of the human sexual response cycle, partner-prescribed sexual perfectionism may contribute in particular to problems at the excitement stage of female sexual function because arousal and lubrication are indicators of excitement. Moreover, persistent and recurrent inability to achieve, or maintain until completion of sexual activity, vaginal lubrication in response to sexual excitement can result in pain during sexual intercourse (Simons & Carey, 2001); and reduced sexual arousal is a diagnostic criterion of Female Sexual Interest/Arousal Disorder (DSM-5; American Psychiatric Association, 2013) which is characterized by a lack of, or significantly reduced, sexual interest/arousal. Furthermore, sexual arousal plays a key role in Basson’s (2001) circular model of female sexual function which features a responsive form of sexual desire that follows sexual arousal (in contrast to the traditional linear model in which sexual arousal follows sexual desire) (cf. Giles & McCabe, 2009). Based on the present findings, partner-prescribed sexual perfectionism could be a psychological factor contributing to Female Sexual Interest/Arousal Disorder and sexual problems caused by reduced lubrication. As to why this is the case, we can only speculate. One possibility is that partner-prescribed sexual perfectionism (thinking that one’s partner expects sex to be perfect) leads to sexual performance anxiety which then negatively affects sexual function (cf. McCabe, 2005). In research on general perfectionism, socially prescribed perfectionism has been linked to performance anxiety in musicians (Kobori, Yoshie, Kudo, & Ohtsuki, 2011), so it is reasonable to assume that partner-prescribed sexual perfectionism too would show a positive relationship with sexual performance anxiety, particularly given the links the present study and Stoeber et al. (2013) found between partner-prescribed sexual perfectionism and general sexual anxiety

The present findings expand on earlier findings on sexual perfectionism and sexual function in two important ways. First, they are the first findings demonstrating a link between sexual perfectionism and female sexual function. Second, they qualify earlier findings linking sexual perfectionism and male sexual function (Eidelson & Epstein, 1982; Quadland, 1980) by suggesting that it is important to differentiate personal and interpersonal aspects in people’s beliefs that one should always perform perfectly during sex. In the present study, such beliefs had no negative effects when they had a personal focus, that is, when they reflected women’s personal standards (self-oriented sexual perfectionism). Only when (1) the beliefs had an interpersonal focus, that is, when they reflected women’s beliefs that *others* expected them to be a perfect sexual partner, and (2) *others* were women’s sexual partners (partner-prescribed sexual perfectionism) did these beliefs have a negative effect on sexual function and sexual self-concept. This was not the case when *others* were society or people in general (socially prescribed sexual perfectionism) which further corroborates Snell’s (1997) conception of multidimensional sexual perfectionism differentiating partner-prescribed and socially prescribed sexual perfectionism.

The importance of differentiating partner-prescribed and socially prescribed sexual perfectionism was also evident in the unique relationships the four forms of sexual perfectionism showed in the cross-sectional analyses. Once the overlap between the forms was statistically controlled, only partner-prescribed sexual perfectionism showed unique relationships with all indicators of sexual function (except satisfaction): negative relationships with desire, arousal, lubrication, and orgasm and a positive relationship with pain during intercourse. Four of these indicators are associated with sexual function disorders in the DSM-5 (American Psychiatric Association, 2013). Low desire and low arousal are indicators associated with Female Sexual Interest/Arousal Disorder (described above). Low orgasmic functioning is an indicator associated with Female Orgasmic Disorder which is characterized by difficulty experiencing orgasm and/or markedly reduced intensity of orgasmic sensations. Pain during intercourse is an indicator associated with Genito-Pelvic Pain/Penetration Disorder which is characterized by marked vulvovaginal or pelvic pain during vaginal intercourse or penetration attempts. In contrast, socially prescribed sexual perfectionism showed no unique relationships with any indicator of female sexual function. However, both partner-prescribed and socially prescribed perfectionism were associated with a negative sexual self-concept as indicated by unique negative relationships with sexual esteem and unique positive relationships with sexual anxiety and sexual problem self-blame.

In comparison, self-oriented and partner-oriented sexual perfectionism emerged as mixed adaptive–maladaptive forms of sexual perfectionism, as was expected from previous findings (Snell, 2001; Snell & Rigdon, 2001; Stoeber et al., 2013). On the one hand, self-oriented sexual perfectionism showed unique positive relationships with sexual esteem and female sexual function regarding desire and arousal, with the positive relationship between self-oriented sexual perfectionism and arousal being stronger in women who had no partner compared to women who had a partner. On the other hand, it showed a unique positive relationship with sexual problem self-blame (i.e., the tendency to blame oneself for sexual problems) indicating a negative sexual self-concept. Partner-oriented sexual perfectionism too showed a positive relationship with sexual esteem. In addition, it showed a negative relationship with sexual anxiety indicating a positive sexual self-concept. Regarding female sexual function, however, partner-oriented sexual perfectionism showed a unique negative relationship with sexual satisfaction indicating that women who have perfectionistic expectations for their sexual partner tend to be less satisfied with the sex they are having compared to women who do not have these expectations.

Sexual satisfaction is an important aspect of female sexual function (Rosen et al., 2000) as many women experience low levels of sexual satisfaction despite having functional levels of desire, arousal, and orgasm (Basson et al., 2001; Dundon & Rellini, 2010). Research has shown that sexual satisfaction is positively associated with overall physical and psychological well-being (e.g., Davison, Bell, LaChina, & Davis, 2009). Furthermore, sexual satisfaction plays an important role in relationship satisfaction, stability, and functioning (e.g., Butzer & Campbell, 2008; Yeh, Lorenz, Wickrama, Conger, & Elder, 2006). Consequently, the finding that partner-oriented sexual perfectionism was the only form of sexual perfectionism that showed a unique negative relationship with sexual satisfaction is noteworthy. Moreover, this finding is in line with findings from research on general perfectionism indicating that other-oriented perfectionism, while associated with a positive self-concept, is a mixed adaptive–maladaptive form of perfectionism associated with interpersonal problems, uncaring-callous personality traits, and a low regard for others (Hewitt & Flett, 2004; Stoeber, 2014a, 2015).

### Limitation and Future Studies

The present study has a number of limitations that should be noted. First, the study was the first to examine whether multidimensional sexual perfectionism predicted longitudinal changes in women’s self-concept and female sexual function, and so was largely exploratory. Hence, future studies need to replicate the longitudinal relationships of partner-prescribed sexual perfectionism before firm conclusions can be drawn regarding the detrimental effects that this form of sexual perfectionism has on women’s sexual well-being. In addition, these studies should include further variables to clarify the mechanisms whereby partner-prescribed sexual perfectionism negatively affects women’s sexual function (e.g., sexual performance anxiety). Furthermore, future studies should differentiate spontaneous and responsive desire (Basson, 2001) and reinvestigate the moderating effect that relationship status (whether women had a partner or not) had on the positive relationship that self-oriented sexual perfectionism showed with arousal. Because the relationship was stronger in women who had no partner, self-oriented sexual perfectionism may show stronger links with spontaneous desire compared to responsive desire.

Second, one-third of the sample did not have a partner (casual or committed relationship, cohabitation, or married/partnered) at Time 1, and so completed the questions regarding partner-oriented and partner-prescribed sexual perfectionism with respect to past or hypothetical sexual partners. There was also substantial attrition from Time 1 to Time 2, particularly among students. This may have been because the Time 2 assessment was from May to June when students were focused on exams (revising for exams in May, taking exams in June). Moreover, participants who responded to both parts of the survey (Time 1 and Time 2) and thus formed the longitudinal sample of the study had lower levels of partner-prescribed sexual perfectionism than participants who responded only to the first part (Time 1). Consequently, it is possible that the longitudinal relationships that partner-prescribed sexual perfectionism showed in the present study only apply to women with lower levels of partner-prescribed sexual perfectionism.

Third, it is unclear to what degree the longitudinal findings are specific to the time interval (3–6 months) the present study examined. This concerns not only the question of whether the present findings would replicate if different intervals were examined, but also the question of whether the size of the relationships would increase and whether the other forms of sexual perfectionism would show longitudinal relationships with longer intervals (e.g., 1 year). Furthermore, it is unclear if the present findings would replicate in clinical samples such as women diagnosed with a sexual function disorder or women seeking treatment for sexual problems. In addition, the majority of women the present study examined were relatively young (see participants section) which is relevant because the idea that sex can be perfect implies a conceptualization of sexual behavior focusing on performance. As the negative correlations we found between sexual perfectionism and age imply, young people may think about sex in a manner that gradually gives way to a different understanding of sex from an activity where you can be perfect (or make mistakes) to an affective experience involving shared pleasures or relationship building.^3^ Consequently, future research may profit from reinvestigating the present findings using longer time intervals and including clinical samples and a greater percentage of women in their forties and fifties to confirm that the findings generalize beyond the samples we examined in the present study.

Fourth, the present study did not include a measure of general perfectionism. Although domain-specific forms of perfectionism have been shown to be better predictors of domain-specific characteristics, processes, and outcomes than general measures of perfectionism (Dunn, Craft, Causgrove Dunn, & Gotwals, 2011; Stoeber & Yang, 2015), future research on sexual perfectionism should include measures of general perfectionism to investigate whether sexual perfectionism explains variance in sexual self-concept and sexual function beyond general perfectionism. In addition, future research may consider including partner’s self-oriented sexual perfectionism (see Footnote 1) to investigate whether the effects of women’s partner-prescribed sexual perfectionism are mitigated by the sense that their partner expects sexual perfection also from himself or herself. Finally, future research may want to investigate the long-term stability of sexual perfectionism. In the present study, sexual perfectionism showed test–retest correlations between .57 and .65 comparable to the 3-month test–retest correlations found for general perfectionism (e.g., Hewitt, Flett, Turnbull-Donovan, & Mikail, 1991) which suggests that individual differences in sexual perfectionism may be as stable over time as individual differences in general perfectionism.

### Conclusions and Clinical Implications

Our study represents the first longitudinal study of multidimensional sexual perfectionism and makes a significant contribution toward a better understanding of the relationships between women’s sexual perfectionism, sexual self-concept, and sexual function. In particular, our finding that partner-prescribed sexual perfectionism predicted longitudinal decreases in female sexual function regarding arousal and lubrication makes an important contribution to the research literature examining potential effects that personality factors have on female sexual function (e.g., Crisp, Vaccaro, Fellner, Kleeman, & Pauls, 2015). Furthermore, the finding that partner-prescribed sexual perfectionism was not only associated with lower sexual esteem and higher sexual anxiety, but predicted longitudinal decreases in sexual esteem and increases in sexual anxiety suggest that partner-prescribed sexual perfectionism is a psychological factor that may contribute to sexual self-concept problems in women. Clinicians, therapists, and counselors working with women reporting sexual self-concept problems and problems with sexual functioning should therefore explore whether partner-prescribed sexual perfectionism—beliefs that their partner imposes perfectionistic standards and expectations on them as a sexual partner—plays a role in these problems. If these beliefs do not reflect veridical perceptions of partners’ actual expectations, these women may benefit from cognitive-behavioral treatment questioning and restructuring these beliefs (Egan, Wade, Shafran, & Antony, 2014) to help them develop a more functional view of their sexuality. However, if they reflect veridical (or partly veridical) perceptions of partners’ actual expectations, couple therapy (e.g., McCarthy, 2002) may be more appropriate.

## Footnotes

1 In Snell’s model, partner-oriented sexual perfectionism is called “partner-directed sexual perfectionism,” and partner-prescribed sexual perfectionism is called “self-directed sexual perfectionism from one’s partner.” Furthermore, the model includes a fifth form of sexual perfectionism called “partner’s self-oriented sexual perfectionism” reflecting people’s beliefs about their sexual partner’s self-oriented sexual perfectionism. This form, however, has no correspondence in previous theory and research on general perfectionism and was therefore disregarded in the present research (see also Stoeber et al., 2013).

2 Details (*M*s, *SD*s, *t* values, *F* values, and correlations) are available from the first author.

3 We are grateful to an anonymous reviewer for this suggestion of age differences in the conceptualization of sex.
